# How Does Consumers’ Perception Influence Their Food Choices?

**DOI:** 10.3390/foods15142448

**Published:** 2026-07-10

**Authors:** Wilma Maria Coelho Araújo, Sandra Fernandes Arruda

**Affiliations:** Department of Nutrition, College of Health Sciences, University of Brasilia, Campus Darcy Ribeiro, Brasilia 70910-900, DF, Brazil; arruda@unb.br

## 1. Introduction

Mazon [1] states that research on consumer behavior originated from studies conducted in the field of psychology (social psychology) and was grounded in constructs such as attitudes, communication, and persuasion, before later expanding to studies that involved other aspects, such as memory, information, and decision-making. Georgesu-Roegen [2] and Greig [3] were precursors to research on consumer behavior, with an emphasis on economic aspects, and such studies remained prominent in the 1950s.

Zwich [4] was the first to include demographic issues as a variable in consumer choice behavior, while Harp [5] included socioeconomic aspects to identify the social relationships that determine consumers’ motivations in food purchasing, according to Mazon [1]. Between the late 1950s and the subsequent decade, Ferber and Wales [6], Katona [7], Howard [8], Newman [9], and Engel et al. [10] investigated the variables influencing consumer choice. Engel et al. [10] pioneered a decision-making process model demonstrating how consumers decide on the purchase, repurchase, or rejection of a product through distinct stages. These investigations were grounded in the premise of describing, understanding, and predicting consumer behavior based on individual motivations [1].

Between the 1980s and the 1990s, research integrated the fields of economics and psychology—giving rise to behavioral economics—and indicated that consumer decision-making is not entirely rational. Specifically, consumers do not always maintain self-control in their choices and are often influenced by demographic and psychographic variables, their surrounding environment, and organizational marketing actions [1].

At the end of the 1990s, Mainieri et al. [11] evaluated how consumers’ beliefs influenced environmental concern, “green” purchasing, and environmental attitudes, while examining the positive correlation between beliefs and gender. Concurrently, Roozen and Pelsmacker [12] investigated consumers’ attitudes towards the “green” value of environmentally friendly aspects across the full consumption cycle (purchase, use, and disposal) [1].

According to Lim et al. [13], key research topics in the field include information processing, consumption communities, consumption value, sustainable consumption, intergenerational consumer behavior, consumer–brand relationships, consumer ethics, and conditional relationships.

Chauí [14] defines perception as a sensory knowledge of configurations that moves from the inside out and is induced by external stimuli. Consequently, it is a process that involves an interaction between the external world and the individual, influencing the formation of beliefs and the understanding of reality.

Food production and processing are as old as human history. Large-scale industrial production began in the 17th century and expanded product distribution, influencing both rural and urban activities. At the end of the 18th century, with the Agricultural Revolution, urban prosperity became strongly dependent on agriculture. This period saw the emergence of critical themes such as waste control, sustainability, and quality—including physical, chemical, sensory, hygienic–sanitary, nutritional, and legal aspects [15].

Carvalho [16] described the profound impact that the Second World War (20th century) had on the European agricultural and food sectors, where infrastructure destruction and workforce led to critical food shortages. This crisis forced European governments to implement emergency actions to boost production, legitimizing direct state intervention to ensure citizen well-being and protect family property.

This period witnessed a technological surge: advancements in chemistry and genetics yielded synthetic fertilizers, herbicides, and pesticides, allowing for the selection of varieties of plant and animal species. Simultaneously, mechanics and engineering introduced advanced mechanization and drainage systems, all driven by the goal of maximizing the food supply for consumers. Alongside these tactics, advancements in scientific knowledge regarding food production, processing, and market regulation fueled the growth of the food industry. As consumers became increasingly aware of the benefits of healthy foods, they began to demand a steady supply of safe and diverse food products from industry [16].

Factors such as food production and distribution systems, shifts in consumer profiles and attitudes toward conscious consumption, and the interdisciplinarity nature of food safety governance, among other aspects, have significantly influenced consumers’ perception of quality in its broadest sense, thereby shaping consumer trust in market products. Although the food industry worldwide follows current consumption trends regarding sensory appeal and pleasure, health and well-being, convenience and practicality, quality and reliability, and sustainability and ethics, consumers remain skeptical about the benefits arising from these production and processing methods [17,18].

These uncertainties have prompted a resurgence in small-scale production, in contrast to conventional large-scale systems, as well as the emergence of niche markets, such as organic foods, and concerns about animal welfare, the environment, and sustainability [18].

Food selection is a voluntary and conscious process of choosing available food substances. It involves the stages of selection, preparation, ingestion, and absorption. In turn, nutrition is the biological process through which the organism assimilates and transforms these substances, converting nutrients into simpler compounds that fulfill physiological roles essential for maintenance, prevention, and the recovery of human health and well-being.

According to Zeithaml [19], perceived product quality is a consumer’s subjective judgment regarding excellence or superiority, shaped by both tangible and intangible attributes like brand, reputation, and communication. Kahneman [20] demonstrates that individual judgment and subsequent decision-making, including but not limited to food choices, rely on two cognitive systems: one based on intuition, emotions, and past experiences, and another guided by logic, reasoning, and available information.

The concept of food quality is shaped by the dynamics of the consumption relationship and involves the State, the productive sector, and consumers. Consequently, it can be analyzed under three distinct categories: (a) the basic level, which includes the physicochemical and safety characteristics that all food products must meet and which are generally regulated by the State; (b) nutritional quality, a critical attribute in consumer evaluation due to its direct health implications; and (c) value attributes, which include sustainability dimensions such as environmental protection, fair labor practices and food traditions, among others [21].

Spers [22] notes that this type of social relationship is characterized by pronounced information asymmetry, particularly regarding the parameters that define food quality. These asymmetries are largely driven by bounded rationality, complexity, and uncertainty surrounding the choice of raw materials, ingredients, additives, products, processes, and formulations. Ultimately, these dynamics manifest as significant disparities in the level of information held by the involved parties: the producer, the consumer, and the State [23].

A heuristic is a mental shortcut that enables quick decision-making and judgment without the need for extensive research or information analysis. While a wide range of heuristics governing human decision-making have been identified, heuristic thinking becomes the predominant mechanism for food choices, particularly when consumers have limited information to evaluate aspects such as food safety or the sustainability impact of their consumption [24].

A systematic review by König et al. [25] describes how emotions, personality traits, and relevant sociocultural factors form the foundation of these heuristics. Emotions are psychological and physiological responses to specific stimuli or situations that often result in a behavioral response, influencing how the benefits and risks of an innovation are evaluated. While emotions are subjective experiences, personality traits represent stable patterns of thought and feeling that characterize an individual’s behavior across different situations and over time, though they may evolve with new experiences. Furthermore, cultural factors and norms—the dynamic beliefs, values, customs, traditions, and practices shared by a society—further influence individuals’ behaviors, preferences, and social interactions. Collectively, these factors drive the heuristics that shape consumer perceptions of objects, products, and events.

A preliminary search conducted in the Scopus database on 15 April 2026, using the keywords “food perception”, “food choice”, “consumer behavior”, “consumer preference”, “consumer attitudes”, and “sensory perception”, identified 67 publications between 2001 and 2026. These documents comprise 88.1% original articles, 10.4% reviews, and 1.5% conference presentations. A significant increase in publications was observed from 2016 onwards, followed by a decline during the COVID-19 pandemic, with research peaking in 2025 at 15 articles. The literature spans a wide range of scientific fields: 46 articles were published in agricultural and biological sciences, 22 in nursing, 11 in psychology, and 9 in biochemistry, genetics, and molecular biology. The United States led in publication volume (13 articles), followed by Germany (7), the United Kingdom (7), and Brazil (5). Regarding specific journals, Food Research International published 11 articles, Appetite, The Journal of Food Science 9, and Foods 2 within the scope of the search criteria. 

Considering the definition proposed by Chaui [14], which describes perception as a sensory knowledge of configurations that operates from the inside out and is induced by external factors, this Special Issue—“How Does Consumers’ Perception Influence Their Food Choices?”—features papers that explore a diverse range of subjective and objective elements influencing consumer perception and decision-making.

Badulescu et al. [26], in the article “Socio-Demographic Correlates of Basic Food Needs: A Maslow’s Hierarchy Analysis”, define nutritional food quality as an essential dimension for satisfying fundamental human needs, closely linked to food security, trust in producers, and emotional and cognitive processes in decision-making. Psychological factors such as high stress, anxiety, or depression are cited as critical influencers. The authors also mention that post-pandemic analyses indicate that consumers’ perceptions of both the intrinsic attributes (e.g., taste, health) and extrinsic attributes (e.g., environmental impact, price) of local foods strongly influence purchasing decisions, reinforcing the link between basic needs and overall well-being.

The study provides a comprehensive analysis of sociodemographic factors, segmentations, and predictors of various consumer habits, perceptions, and food needs. Data were collected using a cross-sectional online questionnaire conducted between October 2021 and June 2022 with 1060 participants, utilizing Maslow’s Hierarchy of Needs scale instrument. The authors reported results concerning sociodemographic factors and predictors related to the fundamental needs of the hierarchy—namely, survival (daily food) and food security (food stocks). The findings indicate that gender, age, and education significantly influence food purchases driven by the need for food security, whereas marital status is a significant factor only for survival-related purchases. Furthermore, each socio-demographic factor emerged as a significant predictor for at least one response category on the Likert scale. The relative influence of each predictor was quantified, providing actionable insights for marketing strategies, such as identifying optimal store locations and adjusting, diversifying, or optimizing product ranges based on the characteristics of specific consumer segments and geographic areas [26].

In the systematic review “Consumers’ Drivers of Perception and Preference of Fermented Food Products and Beverages”, Gschaedler-Mathis et al. [16] assert that food selection is a complex process involving biological and physiological factors, as well as the extrinsic and intrinsic characteristics of the product, and psychological, situational, and sociocultural factors. Furthermore, the fermentation of food and beverages is one of the oldest preservation technologies developed by humankind. Historically, fermented products came to represent one-third of the human diet in their regions of origin and, over time, have gained significant social, cultural, economic, and gastronomic importance [27].

It is now well established that fermented products have greater bioavailability of nutrients, as well as probiotic and prebiotic properties, and are therefore associated with various health benefits for consumers. Considering that consumers are increasingly aware of the relationship between diet and a reduced risk of disease, Gschaedler-Mathis et al. [27] conducted a systematic review that aimed to (i) identify the factors that drive consumer perception and preference for fermented food and beverage products, and (ii) classify the factors that influence the perception and consumption of fermented foods.

An analysis of the 92 papers selected concluded that food choice is driven by a complex interplay of biological, product related, and socio-psychological factors [16]. Among these, intrinsic product characteristics, particularly sensory attributes, were identified as the most-studied drivers of fermented food consumption.

Regarding the appreciation of native ingredients in local gastronomy development, Khamitova et al. [28], in their study “The Use of Local Ingredients in Shaping Tourist Experience: The Case of *Allium ursinum* and Revisit Intention in Rural Destinations of Serbia” examined how *Allium ursinum* (wild garlic) influences tourists’ perceptions of authenticity, satisfaction, and revisit intentions in rural destinations. The study involved 336 participants recruited from tourists who consumed dishes containing wild garlic or fresh ingredients at rural households providing culinary services. It aimed to evaluate how this specific local ingredient contributes to shaping tourists’ perceptions of authenticity and satisfaction in rural destinations, given that *Allium ursinum* was selected due to its cultural symbolism, seasonal availability, and traditional culinary significance.

The study confirms that authentic gastronomic experiences with *Allium ursinum* in Serbia positively influence tourists’ cognitive and affective responses, directly shaping behavioral intentions according to the Stimulus–Organism–Response (SOR) model [28]. These findings demonstrate that such experiences strengthen attachment to local food and enhance the likelihood of future visits. The research was conducted between March and May 2024 using a structured questionnaire based on the SOR framework [28].

Wang et al. [29] studied consumer purchase intentions towards meat produced without preventive antibiotic use. This research is critical because antibiotics in food animals are primarily used to control infectious diseases, stimulate growth, and prevent illness. Consequently, the World Health Organization (WHO) has issued guidelines strongly recommending a complete restriction on the use of medically important antibiotics for disease prevention in the absence of a clinical diagnosis.

The overuse and misuse of antibiotics in food animals have raised significant consumer concerns globally. This growing apprehension has led to the emergence and marketing of animal-derived foods produced without antibiotics. Consequently, the authors examined consumers’ purchase intentions regarding meat produced without the preventive use of antibiotics and the factors influencing these, based on the Theory of Planned Behavior (TPB). The authors’ hypothesis considers that this purchase intention is driven by several factors, including health awareness, confidence in the responsible use of antibiotics in livestock farming, objective knowledge about antibiotics in food-producing animals, subjective knowledge about the preventive use of antibiotics, concerns regarding antibiotic residues and resistant bacteria, and general attitudes towards preventive antibiotic practices [29].

Wang et al. [29] found a strong consumer purchase intention for meat produced without preventive antibiotics, suggesting that restricting preventive antibiotic use aligns with consumer demand. Key drives of this intention include health awareness, confidence in livestock management, and knowledge regarding antibiotic use, with the authors recommending that stakeholders improve communication on the health risks of antibiotic-resistant bacteria [29].

In addition, in the article “Purchase Intention of Healthy Foods: The Determinant Role of Brand Image in the Market of a Developing Country,” Zabalaga-Davila et al. [30] investigated the relationship between brand image and the purchase intention of healthy foods. They examined how variables such as perceived quality and satisfaction influence brand trust and brand loyalty within this relationship. This investigation is timely because, in contemporary consumption contexts, the trend toward a healthy lifestyle has significantly increased the demand for healthy foods. These products, characterized by their nutritional value and health benefits, play a crucial role in preventing diseases and promoting well-being. Consequently, the selection of these products involves extrinsic factors such as brand image, price, packaging, and marketing strategies, including the use of specific colors and prominent information on labels.

The research was conducted with 637 participants (a non-probabilistic convenience sample) via an online survey and indicates no significant direct correlation between brand image (BI) and purchase intention (PI), although BI impacts consumer’s perception of quality (BPQ), brand satisfaction (BS), brand trust (BT), and brand loyalty (BL). While BPQ positively influences BS, BT, and BL, it does not directly impact PI, suggesting that positive brand image and quality foster loyalty, but do not always translate into immediate purchase intention [30].

Dry-cured ham is a high-value meat product with significant consumer acceptance, typically classified according to its region of origin. Its final flavor is influenced by unique regional factors, including climate, geography, pig breed, and specific production methods. In this context, Yu and Gu [31] identified the volatile compounds responsible for the flavor profiles of five varieties of dry-cured ham in their study “Characterization of Aroma-Active Compounds in Five Dry-Cured Hams Based on Electronic Nose and GC-MS-Olfactometry Combined with Odor Description, Intensity, and Hedonic Assessment’. To conduct the analysis, five ham samples from renowned categories were purchased from specialty stores authorized by the China Meat Association in Beijing (October 2024), including Black Hoof Cured Ham (BHCH), Superior-grade Xuan-Zi Ham (SXZH), First-grade Xuan-Zi Ham (FXZH), Liang-Tou-Wu Ham (LTWH), and Fei-Zhong-Wang Ham (FZWH). A multi-method approach was employed, combining Gas Chromatography–Mass Spectrometry–Olfactometry (GC-MS-O) and an Electronic Nose (E-Nose).

A total of 29 aroma-active compounds were identified, with GC-MS-O results showing that intensity increased with concentration, highlighting their significance in defining sensory characteristics. A correlation was established between GC-MS-O results and E-Nose responses, while a study of flavor highlights that unpleasant aromas significantly impact consumer acceptance of dry-cured hams [31].

Ribeiro et al. highlight that palm oil is the leading globally traded vegetable oil, produced primarily in Indonesia, Malaysia, and Thailand, with Brazil holding significant expansion potential as the tenth largest producer. The oil, a major source of carotenoids, vitamin E, and unsaturated fatty acids, is widely used in food industry products, including French fries, margarine, and pizza [32].

The study analyzed the perceptions of crude palm oil (CPO) and refined palm oil (RPO) of 1082 participants, revealing high familiarity with CPO’s nutritional profile despite low regular purchasing rates. While awareness of both oil types is limited, participants showed minimal concern regarding the presence of RPO in food, often failing to identify it on product labels.

From the perspective of food science and technology, food irradiation is recognized as an effective technology for promoting food safety. However, most consumers are unaware of the basic concepts of irradiation, often misinterpreting information and maintaining a negative perception of foods treated with ionizing radiation. Faiad et al. [33] validated the Awareness Scale on Consumption of Irradiated Foods (ASCIF) to assess Argentine consumer knowledge, finding that most are unaware of the benefits of food irradiation. The 31-item, four-factor scale demonstrated psychometric consistency and confirmed its utility in identifying challenges facing irradiated products in Argentina.

## 2. Limitations of Published Studies

As this research provides a holistic view of the intrinsic and extrinsic characteristics affecting food quality and the various factors influencing consumer perception, the authors acknowledge several methodological limitations. The authors recommend longitudinal approaches and larger, representative samples to better understand consumer perceptions of food quality, while emphasizing the need for stakeholder-led educational campaigns. Future research should prioritize robust methodologies, deeper theoretical frameworks, and innovative public health communication strategies.

## 3. Conclusions

Consumer perception of food choices is influenced by an interplay of biological and physiological factors, extrinsic and intrinsic product characteristics, and psychological, situational, and sociocultural elements. These dimensions are estimated through various methods and techniques depending on the research hypothesis. Nutritional value, well-being, convenience, product utilization in specific physiological situations, dietary culture, price, brand trust, and official regulation are key attributes driving purchase intention. The gap resulting from informational asymmetry in the producer–regulator–consumer relationship creates opportunities for the media to disseminate information that often lacks legitimacy. Furthermore, fundamental needs vary significantly across sociodemographic groups, directly impacting purchase behavior. Regarding education, although differences are often only marginally statistically significant, they still shape consumer awareness. In the field of food science and technology, research has historically focused on identifying bioactive compounds or developing technologies to extend shelf life. However, contemporary consumers have become more discerning, demanding quality attributes and health benefits that were previously overlooked.

Studies related to knowledge, understanding, perception, purchase intention, food choices, brand trust, and official regulation are on the rise—a trend that contrasts with the limited availability of research utilizing objective and internationally standardized methods. The research presented in this Special Issue underscores the critical importance of disseminating information grounded in scientific knowledge, as well as the continued appreciation of regional ingredients within their cultural contexts, which supports local sustainability initiatives.
